# The “Med. Department of the University of Iowa,” and the Am. Med. Ass.

**Published:** 1852-06

**Authors:** 


*The "Medical Department of the University of Iowa," and the American Medical Association.*

SOME person or persons assuming to act under the authority of the University of Iowa, sent to the Secretary of the Am. Med. Association, at its meeting at Charleston, a protest against the admission of delegates in that body from Rush Medical College. The ground of their objection was, that the College at Chicago did not charge as high fees as suited the more magnificent views of the gentlemen claiming to speak for that University. This protest was referred to the "committee on the organization of the Association," of which Dr. Isaac Hays, of Philadelphia, was chairman. This reference was made the occasion of a great flourish on the part of this famous University, and in the No. of the *Western Medico-Chirurgical Journal* for July, 1851, the principal of its professors put himself on record in the following pledge:

"To this object we have consecrated our very humble abilities, and with whatever spirit we can utter we *will* unfold the truth and plead our cause at Richmond another year."

Sometime since, the chairman of the committee above referred to, was addressed on the part of the Chicago College, and a desire expressed to meet any and all charges which should be brought against the institution. It was stated in reply that no protest had come before the committee; and this was also the substance of the report made at Richmond, so far as related to that subject. No one was there to bring it up on the part of the Iowa University.

That any one holding a position in a medical school should thus publicly violate his voluntary pledge, may, to those unacquainted with the circumstances, appear surprising. By those who are aware that this "Medical Department of the University of Iowa" is only an *alias* for the concern originally started at St. Charles, and which removed to Laporte, Ia., Rock Island, Ill., Davenport, Iowa, and finally to Keokuk, nothing else was expected. The protest was sent to Charleston, not to be referred to any committee, but as a basis for drumming up students at home; the promise to go to Richmond was made for the same purpose. The pretended charges were fabrications. Not one of the professors of this "Iowa University" dares go before such a committee as that, organized by the American Association, with such charges. The refutation would place them in a position from which they could not escape.

In the meantime the protest has had its desired effect. The Association lent itself, unwittingly in this case, as it has done in several others, to the advancement of personal objects. The school has had all the benefit which it could derive from an industrious circulation of a report that Rush Medical College was to be turned out of the American Medical Association. Whether this has been, or will be, sufficient to save the concern from that contempt which has hitherto driven it from place to place, causing it to assume a new name in each, or whether it will share the fate which seems to have overtaken the *Western Medico-Chirurgical Journal*, a little time will determine.

With regard to the Chicago College, its course on the subject of fees has from the beginning been conservative. Its prices have been higher and its requirements more strict than those of any school coming in direct competition with it. Its fees, after the reduction, were still higher actually than those of Laporte; they are now more than three times higher than those of the University of Michigan.

We claim no merit for this: we think the public interest demands there should be no fees. We shall arrange ours on the basis of the Michigan University at as early a period as shall be found practicable. We shall not deviate from our proposed course from any cause not founded on sound reasoning, or the wants of the profession. D. B.

